# Chondroitin sulfate from fish waste exhibits strong intracellular antioxidant potential

**DOI:** 10.1590/1414-431X2020e10730

**Published:** 2021-07-16

**Authors:** L.H.C. Medeiros, B.M.F. Vasconcelos, M.B. Silva, A.A. Souza-Junior, S.F. Chavante, G.P.V. Andrade

**Affiliations:** 1 Programa de Pós-Graduação em Bioquímica e Biologia Molecular, Departamento de Bioquímica, Universidade Federal do Rio Grande do Norte, Natal, RN, Brasil; 2 Instituto Federal de Educação, Ciência e Tecnologia do Rio Grande do Norte, Parnamirim, RN, Brasil

**Keywords:** Glycosaminoglycans, Chondroitin sulfate, Oreochromis niloticus, Antioxidant, Reactive oxygen species

## Abstract

Chondroitin sulfate (CS) is a type of glycosaminoglycan described as an antioxidant molecule that has been found in animal species such as fish. Tilapia (*Oreochromis niloticus*) represents an eco-friendly source of this compound, since its economical processing generates usable waste, reducing the negative environmental impact. This waste was used for CS extraction, purification, characterization by enzymatic degradation, and evaluation of its antioxidant effect. CS obtained from tilapia presented sulfation mainly at carbon 4 of galactosamine, and it was not cytotoxic at concentrations up to 200 µg/mL. Furthermore, 100 µg/mL of CS from tilapia reduced the levels of reactive oxygen species to 47% of the total intracellular reactive oxygen species level. The ability of CS to chelate metal ions *in vitro* also suggested an ability to react with other pathways that generate oxidative radicals, such as the Haber-Weiss reaction, acting intracellularly in more than one way. Although the role of CS from tilapia remains unclear, the pharmacological effects described herein indicate that CS is a potential molecule for further study of the relationship between the structures and functions of chondroitin sulfates as antioxidants.

## Introduction

Chondroitin sulfate (CS) is an important polysaccharide belonging to the family of glycosaminoglycan (GAGs). It is formed by alternating units of D-glucuronic acid (GlcA) β (1>3) linked to a N-acetyl-D-galactosamine (GalNAc) residue found covalently bound to proteins in the form of a proteoglycan (1). CS chains are assembled in the endoplasmic reticulum and Golgi complex and can contain more than 100 disaccharide units, undergoing several modifications during synthesis (2,3). Sulfation is one of the main modifications of the CS chain, and is usually added onto C-4 and/or C-6 of GalNAc and/or C-2 of GlcA, resulting in different forms of CS.

CS is present in the extracellular matrix of connective tissues, such as cartilage, skin, tendons, and the brain. It can be purified and extracted, showing structural variations according to the type of tissue and animal species used for the extraction (4,5). These structural variations lead to different pharmacological effects, such as the antioxidant effects that can be observed with some types of CS, which are characterized by a reduction in the levels of reactive species within organisms (5,6).

One of the most important groups of reactive species are reactive oxygen species (ROS), especially hydroxyl radicals (OH•‒) and superoxide anion radicals (O_2_•^-^), which are formed during cellular respiration and chemical reactions such as the Haber-Weiss and Fenton reactions (7). In these reactions, metal ions such as ferric ions (Fe^3+^) will react with O_2_•^-^ originating from ferrous ions (Fe^2+^), leading to a reaction with hydrogen peroxide in the cell medium, creating OH• and more Fe^3+^, which restarts the cycle (7). The excess of these radicals in living organisms can be definitive for clinical conditions such as osteoarthritis; therefore, the search for molecules that can act as antioxidants is of great relevance (6,8).

In many countries, various available residual tissues from tilapia (*Oreochromis niloticus*) are produced, such as the viscera including the intestines, kidneys, stomach, spleen, and bladder, which are not normally commercialized and can act in processes such as the eutrophication of water reservoirs, causing damage to natural ecosystems (9,10). Thus, the objective of this study was to characterize the CS extracted from tilapia viscera and evaluate its antioxidant potential. Good results obtained herein show that tilapia could be an alternative source for CS extraction, reducing environmental problems related to tilapia cultivation.

## Material and Methods

GAGs were isolated and purified from adult tilapia viscera after proteolysis, Lewatit ion exchange resin treatment, and acetone fractionation (0.5:1.0 v/v; 0.6:1.0 v/v; 0.7:1.0 v/v; 0.8:1.0 v/v; 1.0:1.0 v/v) as previously described (11). Then, the CS from tilapia was purified after fractionation by ion-exchange chromatography with Diethylaminoethyl-Sephacel (DEAE-Sephacel) eluting with 0.8 M NaCl.

The obtained CS was characterized by enzymatic degradation with chondroitinase AC (12), and the cytotoxic effects of the compound extracted from tilapia was verified by the 3-[4,5-dimethylthiazol-2-yl]-2,5-diphenyltetrazolium (MTT) test. RAW 264.7 cells (murine macrophages) were cultured in Dulbecco's modified Eagle's medium (DMEM) supplemented with 10% fetal bovine serum, streptomycin, and penicillin. The plates were grown in a 5% CO_2_ oven at 37°C during the adherence period, followed by removal of the medium and washing with phosphate-buffered saline (PBS). Then, the cells were treated with the samples at concentrations of 20, 40, 100, 200, 400, 1000, and 2000 µg/mL for 24 h. The medium was discarded, and 100 µL of medium containing MTT (5 mg/mL) was added to each well. The plates were then incubated for 4 h, the supernatant was removed, 100 µL of ethanol was added, and spectroscopic readings were performed at 570 nm (Epoch microplate spectrophotometer, Biotek Instruments Inc., USA).

For antioxidant tests, concentrations of the samples that did not show cytotoxicity (20, 40, 100, and 200 µg/mL) were used. Intracellular ROS inhibition tests were performed with the 2,7-dichlorofluorescein diacetate (DCFH-DA) test (13). RAW 264.7 cells were washed and stressed with lipopolysaccharide (LPS), and the samples were applied in their corresponding wells. Hydroxyl radical scavenging was assessed using the salicylic acid-ethanol method *in vitro* (14), superoxide anion radical scavenging was determined by the nitroblue tetrazolium reduction method *in vitro* (15), and ferrous ion chelation was evaluated by the detection of ferrocene *in vitro* (16). For comparison of the effects, these tests were performed at the same concentrations with CS obtained from bovine tracheal tissue (CS_bovine_), which was obtained from Sigma Aldrich (USA).

For statistical analysis, GraphPad Prism software, version 6.0 (GraphPad Software Inc., USA) was used to determine significant differences between treatments by the two-way univariate analysis of variance (two-way ANOVA) because there were more than two independent variables. For all analyses, P values <0.05 were considered statistically significant.

## Results

**Figure 1:**
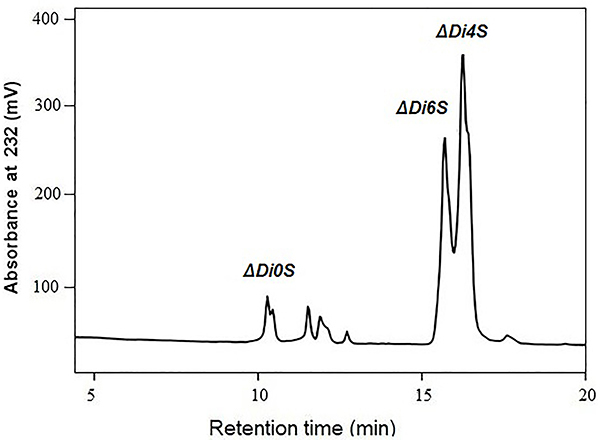
Enzymatic degradation of chondroitin sulfate from tilapia viscera. ΔDi0S: non-sulfated disaccharide; ΔDi4S: disaccharide with sulfation at carbon 4 of galactosamine; ΔDi6S: disaccharide with sulfation at carbon 6 of galactosamine.

Analyses of enzymatic degradation products with chondroitinase AC revealed a prevalence of 59.6% disaccharides containing a sulfate moiety on carbon 4 of galactosamine (ΔDi4S), 36.6% disaccharides sulfated on carbon 6 of galactosamine (ΔDi6S), and 3.4% non-sulfated disaccharide units (ΔDi0S), as seen in Figure 1. A higher prevalence of ΔDi4S characterizes this tilapia GAG as CS type A; thus, the purified compound was named tilapia chondroitin sulfate A (CS-A_tilapia_).

**Figure 2:**
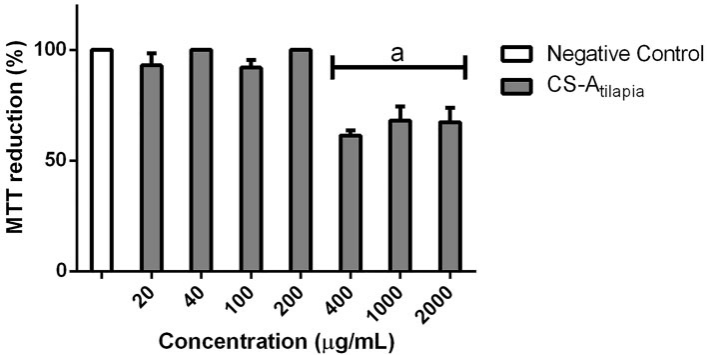
Evaluation of the cytotoxicity in RAW 264.7 cell culture. CS-A_tilapia_: tilapia chondroitin sulfate; Negative control: cells + culture medium. Data are reported as mean±SE. ^a^P<0.01 compared to negative control by ANOVA.

CS-A_tilapia_ did not show cytotoxicity to the treated cells using the MTT assay until the concentration reached 200 µg/mL, maintaining cell survival close to 95% (Figure 2). For this reason, CS-A_tilapia_ could be tested in antioxidant assays, and it was determined that the experiments should focus on concentrations with low cytotoxic effects in RAW cells.

**Figure 3:**
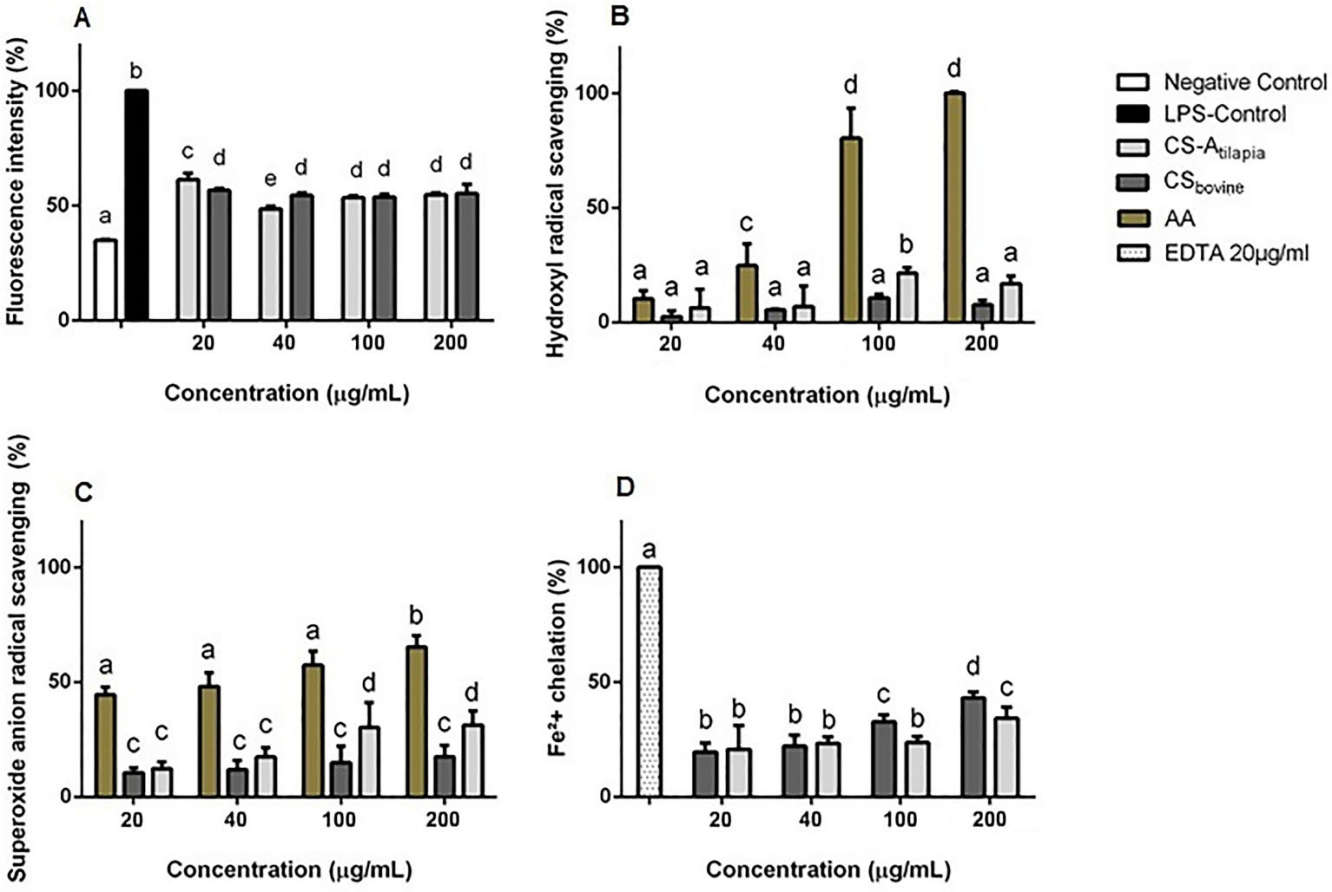
Antioxidant properties of chondroitin sulfate from tilapia viscera. CS-A_tilapia_: tilapia chondroitin sulfate; CS_bovine_: bovine chondroitin sulfate; Negative Control: cells and culture medium; LPS-Control: cells treated with lipopolysaccharide; AA: ascorbic acid. **A**, Intracellular reactive oxygen species inhibition tests. **B**, Hydroxyl radical scavenging. **C**, Superoxide anion radical scavenging. **D**, Ferrous ion chelation. Data are reported as mean±SE. Different letters indicate significant differences by ANOVA (P<0.05).

The total intracellular ROS inhibition test showed that both CS-A_tilapia_ and CS_bovine_ were able to significantly reduce the amount of ROS in the system at all concentrations (P<0.01). CS-A_tilapia_ demonstrated a greater effect at a concentration of 40 µg/mL, reaching 52% of the total inhibition (Figure 3A). Considering that hydroxyl radicals and superoxide anions are the intracellular ROS implicated in cell damage, we next investigated the effects of CS-A_tilapia_ on these ROS.

For the hydroxyl radical and superoxide anion radical scavenging tests, CS-A_tilapia_ and CS_bovine_ showed low effects compared to those of ascorbic acid. However, at a concentration of 100 µg/mL, CS-A_tilapia_ showed a greater capacity for hydroxyl radical scavenging (21%) than CS_bovine_ (10%) (P<0.05) (Figure 3B), and a 30% superoxide anion scavenging by CS-A_tilapia_ *vs* 14% scavenging by CS_bovine_ were observed at this same concentration (P<0.05) (Figure 3C).

These results demonstrated the direct effects of CS-A_tilapia_ on reactive species and raised questions about its use in the Haber-Weiss pathway, which is responsible for forming oxidative species from reactions with metallic molecules, such as iron.

The highest amount of ferrous ion chelation by CS-A_tilapia_ and CS_bovine_ was observed at 200 µg/mL, reaching a maximum of 34 and 43% inhibition, respectively, compared with EDTA, which was assigned as 100% chelation (Figure 3D).

## Discussion

Reports on natural CS obtained from waste that is capable of ROS inhibition are scarce in the literature, although much evidence about the antioxidant potential of CS species has been reported (5,6). For this reason, there is great interest in expanding studies on the role of CS in these physiological events. In this work, an extraction procedure similar to those used previously to obtain compounds with pharmacological potential in different invertebrates and marine species was performed, showing efficiency and reproducibility (11,17). The characterized CS-A_tilapia_, containing 59.6% disaccharides sulfated at position 4 of galactosamine, has been described for the first time.

This structure is reported to have high antioxidant activity, and it is quite similar to the standard CS_bovine_, which is currently among the most commercialized CS compounds (5). This result suggests that these two compounds have similar structures and effects, denoting an alternative eco-friendly source of CS. In addition, CS-A_tilapia_ did not exhibit cytotoxicity until it reached a concentration of 200 µg/mL, which allowed its safe use and helped to define an appropriate dose for antioxidant studies using CS-A_tilapia_. This result is in agreement with a study showing the non-cytotoxicity of CS from shark cartilage (13).

The inhibitory effect of the total intracellular ROS (DCFH-DA test) by CS-A_tilapia_ was more efficient than other polysaccharides found in the literature, and similar effects were obtained with low concentrations of CS-A_tilapia_(13,18). These data are important to strengthen the hypothesis of the safe and efficient use of CS-A_tilapia_ as an antioxidant and could be correlated during the treatment of oxidant stress diseases such as osteoarthritis and Alzheimer's disease (6,7,13).

Low antioxidant activity is common among different sources of CS compared to chemical compounds, such as EDTA and ascorbic acid (5). However, CS-A_tilapia_ exhibited a doubled effect compared to CS_bovine_ at the same concentrations in the hydroxyl and superoxide radical tests, possibly reflecting the ability of CS-A_tilapia_ to donate protons. Furthermore, Fe^2+^ inhibition increased at higher concentrations tested for both CS samples. This effect is due to the ability of the CS molecule to bind metallic molecules in cellular medium, and it is usually concentration-dependent, as previously observed for higher doses of polysaccharides (5,19).

Taken together, these data suggest that the elimination of reactive species and the inhibition of oxidative radical-producing pathways are possibly not the main antioxidant mechanisms of these molecules, suggesting the participation of CS in other signaling pathways (20).

In conclusion, our results helped to understand the structure of CS-A_tilapia_ and suggest it has potential effects as an antioxidant molecule. In addition, the use of tilapia viscera as a source of CS-type molecules after extraction opens a new perspective of minimizing the environmental impact generated by the tailings discarded by tilapia culture, contributing to the sustainability of this economic activity around the world (10).
